# Effect of low-dose exogenous surfactant on infants with acute respiratory distress syndrome after cardiac surgery: a retrospective analysis

**DOI:** 10.1186/s12890-020-01251-2

**Published:** 2020-08-06

**Authors:** Rongyuan Zhang, Xu Wang, Shoujun Li, Jun Yan

**Affiliations:** 1 Department of Pediatric Intensive Care Unit, National Center for Cardiovascular Disease and Fuwai Hospital, Chinese Academy of Medical Sciences, Peking Union Medical College, Beijing, People’s Republic of China; 2 Department of Surgery, Pediatric Cardiac Center, National Center for Cardiovascular Disease and Fuwai Hospital, Chinese Academy of Medical Sciences, Peking Union Medical College, Beijing, People’s Republic of China

**Keywords:** Infants, Pulmonary surfactant, ARDS, Cardiac surgery, Congenital heart disease

## Abstract

**Background:**

Acute respiratory distress syndrome (ARDS) in infants undergoing cardiac surgery is associated with significant mortality and prolonged ventilation; surfactant administration may be a useful therapy. The purpose of this study is to evaluate the effect of low-dose exogenous surfactant therapy on infants suffering ARDS after cardiac surgery.

**Methods:**

We conducted a case-control study of infants diagnosed with moderate-to-severe ARDS (PaO_2_/FiO_2_ < 150) after cardiac surgery. A case was defined as a patient that received surfactant and standard therapy, while a control was defined as a patient that underwent standard therapy. The primary endpoint was the improvement in oxygenation index (OI) after 24-h of surfactant treatment; and secondary endpoints were the ventilator time and PICU time.

**Results:**

Twenty-two infants treated with surfactant were matched with 22 controls. Early low-dose (20 mg/kg) surfactant treatment was associated with improved outcomes. After surfactant administration for 24-h, the surfactant group was much better compared with the control group at the 24-h in OI (difference in average change from baseline, − 6.7 [95% CI, − 9.3 to − 4.1]) (*P* < 0.01) and ventilation index (VI, mean difference, − 11.9 [95% CI, − 18.1 to − 5.7]) (*P* < 0.01). Ventilation time and PICU time were significantly shorter in the surfactant group compared with the control group (133.6 h ± 27.2 vs 218.4 h ± 28.7, *P* < 0.01; 10.7d ± 5.1 vs 17.5d ± 6.8, *P* < 0.01). Infants in the surfactant group under 3 months benefit more from OI and VI than the infants over 3 months in a preliminary exploratory analysis.

**Conclusions:**

In infants with moderate-to-severe ARDS after cardiac surgery, early low-dose exogenous surfactant treatment could prominently improve oxygenation and reduce mechanical ventilation time and PICU time. Infants younger than 3 months may get more benefit of oxygenation than the older ones. Randomized controlled trials are needed to explore the effect of surfactant to ARDS of cardiac surgical infants.

## Background

ARDS is recognized as a life-threatening respiratory complication in infants undergoing cardiac surgery and CPB, associated with significant mortality and prolonged ventilation. Despite many efforts has been made to improve the survival rate, such as lung protective ventilation, fluid management, neuromuscular blockade, prone positioning, inhaled pulmonary vasodilators, and extracorporeal membrane oxygenation, mortality rate from ARDS remains 30–45% [1]. Infants with CHD are more prone to CPB-ARDS, and severe lung injury may impair both lung mechanics and gas exchange [2].

Exogenous surfactant therapy is a routine therapeutic choice for pre-term newborns suffering respiratory distress syndrome. It is suggested that there may be expanded use of surfactant replacement for diseases such as meconium aspiration syndrome [3], pneumonia [4], bronchopulmonary dysplasia [5], and ARDS [6]. Surfactant replacement is an effective and safe therapy by rapidly improving oxygenation and mechanics, and it can improve lung physiology and clinical outcome [6]. Clinical trials reported that exogenous surfactant therapy could be beneficial for children and infants with ARDS without significant adverse effects [7].

The qualitative and quantitative changes in alveolar surfactant of patients with ARDS have been proven [7–9]. Similarly, due to endothelial cells damage, all post-cardiac surgery infants who had ARDS suffered surfactant dysfunction, destruction and inactivation [10–12]. Yet, limited study [13] showed preliminary therapeutic effect of surfactant therapy on these patients, especially, the association between age or drug dose and the efficacy of surfactant therapy. Therefore, this study will focus on: 1 evaluating whether low-dose surfactant therapy would improve oxygenation in infants who had ARDS after cardiac surgery with CPB; 2 trying to identify the most beneficial age of surfactant therapy.

## Methods

### Study design and patients

This is a retrospective, observational, case-control analysis, conducted from January 2015 to June 2019. Ethical Committee of Fuwai Cardiovascular Hospital approved the protocol (Approval NO. 2015–682), and written informed consent was obtained from all participants’ parents.

The study was conducted in a PICU (40 beds) at a 1521-bedded tertiary medical care center in China. Patients entry criteria included: (1) less than 1 year old; (2) complete repair of CHD with CPB; (3) PaO_2_/FiO_2_ lowered than 150 and had been mechanically ventilated for more than 48 h. Exclusion criteria: (1) residual cardiac malformation that must be treated surgically; (2) ECMO; (3) cardiopulmonary resuscitation; (4) airway anomalies that will delay extubation; (5) ejection fracture < 45% (every patient received an ECHO when ARDS was diagnosed); (6) left atrial pressure > 12 (Left atrial pressure of every infant was measured by placing a special catheter into the right atrium then punching through the interatrial septum).

There were 2 different strategies to deal with these infants in our center. Some patients preferred to adopt surfactant, who met the criteria. Other patients preferred to use standard treatment, for worrying about the potential complications such as airway obstruction, or for disliking extra economic burden. Everyone was offered surfactant at the beginning, but surfactant is not a part of our standard treatment protocol.

A total of 7569 children that had cardiac surgery were admitted to PICU, and 3414 of them were infants. 343 infants used mechanical ventilation above 2 days. 78 infants were diagnosed with moderate-to-severe ARDS (PaO_2_/FiO_2_), and who matched inclusion and exclusion criteria. 22 infants who received surfactant in addition to standard care constituted surfactant group. In order to minimize potential bias caused by differences in baseline characteristics between groups, patients were matched in a 1:1 ratio using the following baseline features: age (±30d), weight (±3 kg), RACHS-1, and initial PaO_2_/FiO_2_ (±10). These 22 controls were also offered surfactant but declined (high cost 5, guardians’ preference to use standard treatment 5, and guardians’ refusal for worrying about the potential complications 12). It was a comparative study evaluating the changes in clinical status and outcome between the two groups. The primary endpoint was the improvement in OI after 24-h of surfactant treatment; and secondary endpoints were ventilator time and PICU time.

### Study drug

Surfactant (Calf Pulmonary Surfactant for injection, produced by Shuang he Inc., Beijing, CN) is a modified natural lung surfactant. It is produced by extracting the phospholipids, cholesterol, triglycerides, free fatty acids, surfactant protein B and surfactant protein C from bovine lung surfactant of newborn calf lungs. China Food and Drug Administration approved surfactant for neonatal respiratory distress syndrome.

The standard dose of surfactant is 50-100 mg/kg [14, 15], some patients may have side effects such as intense instant hemodynamic fluctuation and hypoxemia. Due to the high cost and the concern for airway obstruction, dose selection for the infant undergoing cardiac surgery was cautious. Considering that the native surfactant system of these infants was nearly normal before operation, and self-repairing systems of epithelial cells could replenish surfactant after CPB, a low dose of 20 mg/kg nature surfactant as the treatment dosage was used.

### Study intervention

All operations were performed by 2 senior surgeons. Patients in both groups had received standard care according to the hospital protocol. The basic care contained fluid resuscitation, enteral feeds and pain management, and other treatments including cardiac, diuretic, anti-inflammatory. Vital signs, oximetry and hemodynamic parameters would be continuously monitored. The cardiac functions and circulatory blood volume status were obtained by pumping multiple vasoactive agents mainly including catecholamine drugs and giving adequate fluid supplement. Rescue protocol for any severe hemodynamic fluctuation would be prepared. Sedation and mechanical ventilation treatment would be strictly controlled. Supportive management and antibiotics were given as per unit policy. ARDS was diagnosed based on the standard recommended by the North American-European Consensus Conference Committee [16]. The diagnosis of ARDS was confirmed by clinical, radiological and laboratory findings.

Lung protective ventilation strategy was applied to all infants before enrollment. All infants were intubated and supported by mechanical ventilation with synchronized intermittent mandatory ventilation mode of the ventilator (PB 840®). Ventilator settings were adjusted to arterial blood gas results. The peak inspiratory pressure was adjusted to reach a tidal volume goal of 6 ml/kg to 8 ml/kg. To keep the PaCO_2_ below 45 mmHg, the inspiratory time would be set at approximately 0.5 s, with respiratory rate 25–40/min, PEEP 4–8 cmH_2_O. Also, to maintain arterial oxygen saturation above 85% and PaO_2_ above 50 mmHg, peak inspiratory pressure and FiO_2_ needed to be adjusted.

Natural surfactant (bovine) would be given 20 mg/Kg (35 mg/ml). After receiving the written parental permission, surfactant would be instilled into the trachea via an endotracheal tube using a small catheter in 4 equal aliquots, which would be instilled in four different positions (left, head up then down, right). Manual ventilation with 100% O_2_ would be applied for 5 min after the treatment. With concomitant sedation and muscle relaxation, the next tracheal suctioning would be performed at least 4 h later. The acute effects of surfactant therapy would be evaluated 24- h after the treatment. Vital signs were monitored continuously and recorded for 60 min after the intervention. Chest radiographs were acquired before and after surfactant administration every day.

### Data collection

Data was entered on a pre-designed case record form from the patients’ archived files. The data extracted included patient demographics, blood gases, ventilator settings, complication, total time on ventilator, total time in PICU and clinical outcomes. Ventilator days were counted from the first day that a patient received mechanical ventilation. Ventilator parameters were recorded before the start of surfactant administration. After surfactant treatment, OI and VI were derived from the measured data. OI was calculated via mean airway pressure * FiO_2_ *100/PaO_2_ and VI was calculated via PaCO_2_ * peak inspiratory pressure * respiratory rate/1000.

The baseline demographic and clinical characteristics that were collected were age, weight, sex, RACHS-1, total on-pump time, aortic clamping time, OI, VI, PaO_2_/FiO_2_, PaO_2_ and the status of the patient within the time of inclusion (Table 1). In addition, the severity of illness at the time of inclusion was recorded and assessed by using the SOFA score [17]. Moreover, the daily vital signs, urine output, laboratory data, ventilator settings, vasopressor dosage were extracted.

**Table 1:** Study population and baseline characteristics

Characteristic	Surfactant *n* = 22	Control *n* = 22	*P* Value
Age (months)	5.5(±1.6)	5.3(±1.8)	0.698
Weight (kg)	6.2(±1.2)	6.3(±1.5)	0.808
Sex (male/female)	13/9	12/10	0.585
RACHS-1 II (%)	7(31.8)	7(31.8)	
RACHS-1 III (%)	8(36.3)	8(36.3)	
RACHS-1 IV (%)	7(31.8)	7(31.8)	
Total on-pump time (min)	146.8(±44.5)	138.4(±35.8)	0.382
Aortic clamping time (min)	57.3(±18.1)	52.3(±15.2)	0.326
Time of inclusion (days after operation)	3.0(±1.6)	2.9(±1.4)	0.826
OI	13.9(±3.8)	13.2(±3.2)	0.512
VI	42.4(±7.6)	40.6(±8.4)	0.460
PaO_2_/FiO_2_	88.5(±15.4)	90.3(±17.5)	0.719
PaCO_2_	43.0(±5.8)	45.8(±7.5)	0.173
SOFA score	10.5(±2.9)	11.2(±3.1)	0.325
Use of neuromuscular blockers (%)	12(54.5)	14(63.6)	0.106
Vasopressor use (%)	22(100)	22(100)	
Body temperature (°C)	36.5(±0.4)	36.4(±0.5)	0.468
Mean arterial pressure (mmHg)	44.6(±18.4)	47.2(±15.6)	0.616
White cell count (10^9^/L)	13.4(±4.2)	12.1(±3.8)	0.288
CRP (mg/L)	58.6(±20.7)	49.2(±16.1)	0.100
Latate (mmol/L)	1.1(±0.4)	0.8(±0.3)	0.013
Creatinine (umol/L)	64.8(±32.2)	72.1(±20.5)	0.375
Clinical pneumonia+ CPB injury (%)	10(45.5)	12(54.5)	0.818
Proved pneumonia (%)	7(31.8)	9(40.9)	0.358
Viral, non-RSV (%)	2(9)	3(13.6)	0.000
RSV (%)	1(4.5)	2(9)	0.000
Bacterial (%)	3(13.6)	4(18)	0.003
CPB injury (%)	12(54.5)	10(45.5)	0.818

Surfactant: patients who had ARDS after cardiac surgery received standard treatment plus exogenous surfactant. Control: patients who had ARDS after cardiac surgery received standard treatment only. The data are presented as mean ± standard; qualitative data are presented as numbers (%)

*RACHS-1* risk adjustment congenital heart surgery-1, *CPB* cardiopulmonary bypass, *VI* ventilation index, *OI* oxygenation index

### Statistical analysis

Qualitative data were presented as frequencies and percentages, whereas quantitative data were presented as mean, standard deviation. The unpaired t-test was used for comparison between patients in surfactant group and control group. Classification data number (percentage) aggregation, and Chi-square or Fisher’s exact test. The cumulative percentages of extubated patients were analyzed using Kaplan-Meier survival analysis with the log-rank test. The data was analyzed using SPSS version 20.0. The *p*-value of < 0.05 was considered as statistically significant.

## Results

### Patient characteristics

Detailed demographic information of infants in surfactant group and control group are listed in Table 1. There was no significant difference in age, weight, sex, RACHS-1, total on-pump time and aortic clamping time between the surfactant group and the control group. There was no significant difference in OI, VI, PaO_2_/FiO_2_ and PaCO_2_ between the two groups at the beginning (Table 1). The mean time of surfactant administration was 3 days after operation.

### Clinical usefulness of low-dose exogenous surfactant

**Fig. 1:**
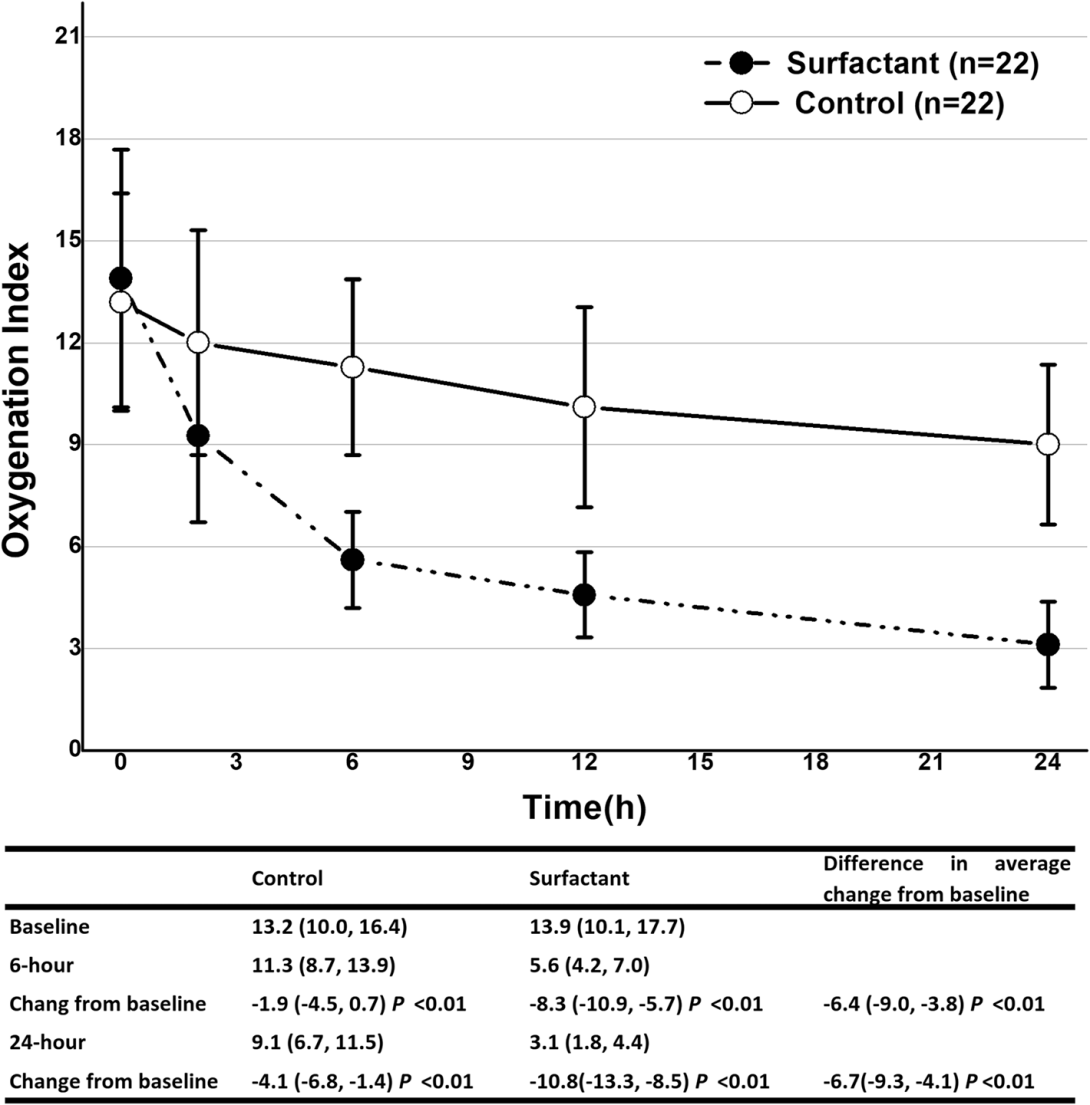
Change of Oxygenation Index (OI) before and after surfactant treatment compared with the control

**Fig. 2:**
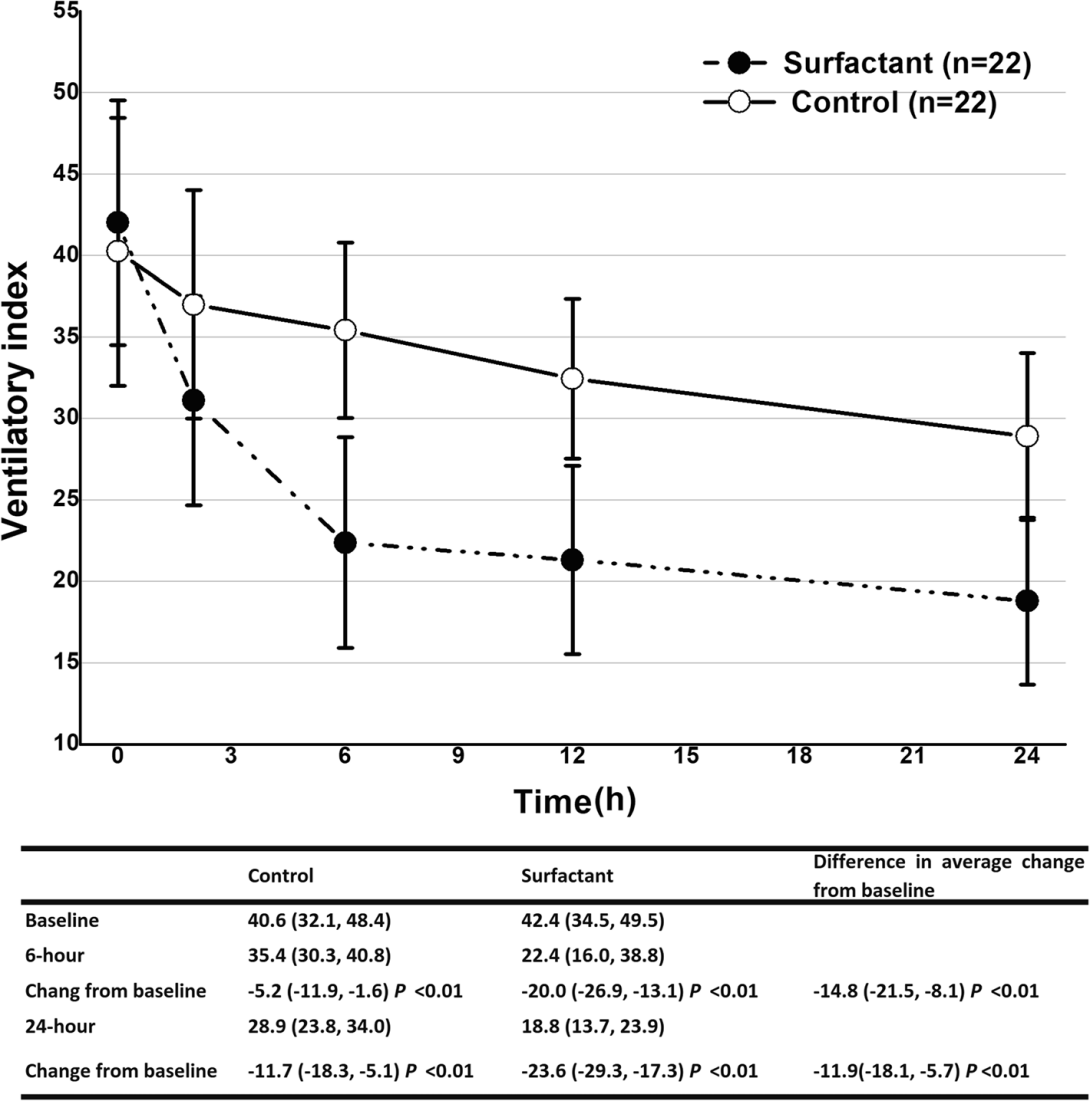
Change of Ventilatory Index (VI) before and after surfactant treatment compared with the control

**Fig. 3:**
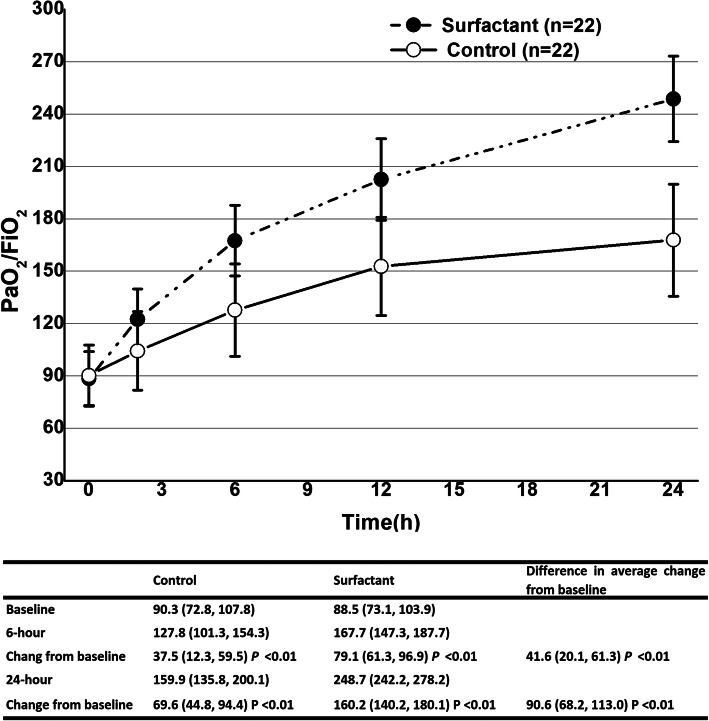
Changes of PaO_2_/FiO_2_ before and after surfactant treatment compared with the control. FiO_2_ = fraction of inspired oxygen, PaO_2_ = arterial partial pressure of oxygen

**Fig. 4:**
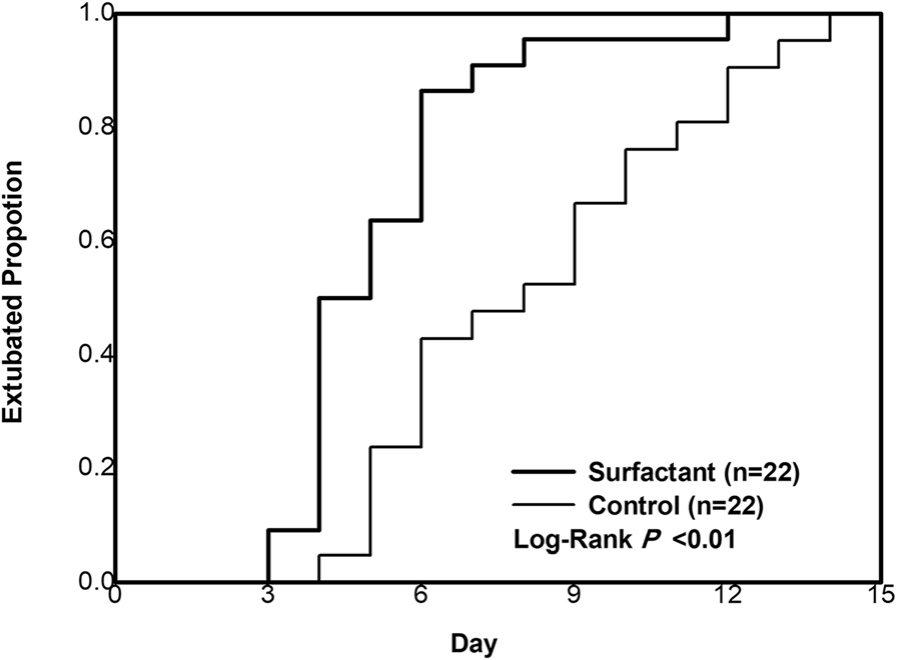
Extubated proportion of surfactant compared with control

The treatment was well tolerated as well. No side effects and complications were observed during surfactant treatment. Compared with the control group, infants of the surfactant group showed better improvement in OI, VI and PaO_2_/FiO_2_ ratio. All variables before and after surfactant therapy is given in Table 2 and Figs. 1, 2 and 3. Infants receiving surfactant had significantly shorter ventilation time and PICU time (Table 2). All patients were extubated within 15 days. Infants in surfactant group were extubated earlier than control group (*P* < 0.01) (Fig. 4).

**Table 2:** Clinical outcomes (in 30 days)

Characteristic	Surfactant *n* = 22	Control *n* = 22	*P* Value
Alive/death (n/n)	22/0	22/0	–
Complication			
Pneumothorax (%)	2(9)	5(22.7)	0.001
Digestive tract hemorrhage (%)	0(0)	1(4.5)	0.000
Rescue treatment (%)			
CPAP (%)	3(13.6)	8(36.3)	0.015
Re tracheal intubation (%)	1(4.5)	2(9)	0.000
PD (%)	1(4.5)	2(9)	0.000
HFOV (%)	0(0)	1(4.5)	0.000
Total time on ventilator (h)	133.6 ± 27.2	218.4 ± 28.7	0.000
Total time in PICU (day)	10.7 ± 5.1	17.5 ± 6.8	0.001

Surfactant: patients who had ARDS after cardiac surgery received standard treatment plus exogenous surfactant. Control: patients who had ARDS after cardiac surgery received standard treatment only. The data are presented as mean ± standard; qualitative data are presented as numbers (%)

*CPAP* treatment with continuous positive airway pressure, *PD* treatment with peritoneal dialysis, *HFOV* treatment with high frequency oscillatory ventilator

The surfactant group was better compared with the control group at the 6-h with respect to both OI (difference in average change from baseline, − 6.4 [95% CI, − 9.0 to − 3.8]) (*P* < 0.01) and VI (mean difference, − 14.8 [95% CI, − 21.5 to − 8.1]) (*P* < 0.01). At the 24-h follow-up, the change from baseline in OI (difference, − 6.7 [95% CI, − 9.3 to − 4.1]) (*P* < 0.01) and VI (mean difference,-11.9 [95% CI, − 18.1 to − 5.7]) (*P* < 0.01) were much better in the surfactant group than the control group (*P* < 0.01). (Figs. 1 and 2.

Similarly, compared with the control group, surfactant treatment was associated with greater improvement (*P* < 0.01) at 6-h in PaO_2_/FiO_2_ (mean difference, 41.6 [95% CI, 20.1 to 63.1]) (*P* < 0.01). At the 24-h follow-up, the change from baseline in PaO_2_/FiO_2_ (mean difference, 90.6 [95% CI, 68.2 to 113.0]) (*P* < 0.01) was much higher in the surfactant group than the control group (Fig. 3).

None of the patients showed significant clinical deterioration and changes in hemodynamic status and other vital signs with the application of surfactant (Table 2). Five patients (22.7%) in control group and 2 patients (9.1%) in surfactant group had pneumothorax; and 1 (4.5%) patient in control group had digestive hemorrhage, none happened in surfactant group (*P* < 0.01). Three patients (13.6%) in surfactant group and 8 (36.3%) in control group needed CPAP after extubation (*P* < 0.01). There were 2(9%) and 1(4.5%) patients in control group and surfactant group needing retracheal intubation and peritoneal dialysis respectively. One (4.5%) patient in control group needed HFOV, none happened in surfactant group (*P* < 0.01).

### Exploratory subgroup analysis

The 22 patients in surfactant group were assigned to younger than 3 months subgroup (10 infants) and older than 3 months subgroup (12 infants). RACHS-1, total on-pump time and aortic clamping time of the two subgroups were similar (*P* < 0.01) (Table 3). For the subgroups based on age have very low numbers, preliminary exploratory analysis showed: surfactant showed better efficacy in OI and VI in the less than 3 months subgroup (Table 4). There was no statistical difference in ventilator time and PICU time between the two subgroups (Table 3).

**Table 3:** Baseline characteristics and clinical outcomes of subgroups of surfactant group

Characteristic	≤3 months subgroup *n* = 10	> 3 months subgroup *n* = 12	*P* Value
Age (months)	3.7 ± 0.8	7.0 ± 2.1	0.000
Weight (kg)	5.3 ± 1.2	7.3 ± 2.2	0.018
Sex (male/female)	6/4	7/5	0.671
RACHS-1 II (%)	3 (30)	4 (33)	0.228
RACHS-1 III (%)	4 (40)	4 (33)	0.387
RACHS-1 IV (%)	3 (30)	4 (33)	0.228
Total on-pump time (min)	155.2 ± 43.6	146.9 ± 46.7	0.673
Aortic clamping time (min)	52.0 ± 19.4	49.6 ± 20.6	0.782
Ventilation time (h)	140.2 ± 32.8	123.6 ± 47.5	0.361
PICU time (day)	12.8 ± 4.2	10.7 ± 4.0	0.244

The data are presented as mean ± standard; qualitative data are presented as numbers (%)

**Table 4:** Oxygenation index and ventilation index of subgroup in surfactant group (in 30 days)

Characteristic	≤3 months subgroup *n* = 10	> 3 months subgroup *n* = 12	Difference in average change from baseline
Baseline of OI	16.3(13.2, 19.4)	11.9 (7.9, 15.9)	–
24-h	3.7(3.0, 4.4)	2.9 (1.6, 4.2)	–
Change from baseline	12.6 (8.8, 16.4)	9.0 (7.6, 10.4)	3.6 (1, 6.2) *P* < 0.01
Baseline of VI	49.2 (41.3, 57.1)	39.6 (33.3, 45.9)	–
24-h	21.2 (17.0, 25.4)	19.4 (16.2, 22.6)	–
Change from baseline	28.0 (21.9, 34.1)	10.1 (5.3, 15.9)	17.9 (12.2, 23.4) *P* < 0.01

Values are presented as mean (95% CI)

*OI* oxygenation index, *VI* ventilation index

## Discussion

In this study, with the treatment of low dose surfactant, rapid improvement in oxygenation, and significant decrease in duration of ventilator time and PICU time were observed. This is the first study about low-dose surfactant therapy in infants after cardiac surgery with CPB. Preliminary exploratory analysis showed infants younger than 3 months benefited more from oxygenation improvement than the infants older than 3 months.

The pathophysiology of ARDS following CPB has not been completely defined yet. Inflammatory response induced by CPB could be exacerbated by accumulation of cytokines, and infants seem to be less susceptible to this response [18]. Meanwhile, prolonged mechanical ventilation can worsen ARDS by damaging the alveolocapillary barrier in the lungs [19]. The reduced compliance and a ventilation-perfusion mismatch hinted that the surfactant system was damaged [20, 21]. Nearly one third of post-cardiac surgery infants have been found surfactant components changes [22]. Exogenous surfactant may contribute in preventing the surfactant system from developing extreme disturbance.

Respiratory tract infections are the most common risk factors of ARDS in infants, and CPB is a risk factor reported in cardiac patients. Around 50% of patients in each group had clinical courses complicated by pneumonia, infants with unrepaired CHD often suffer from malnutrition and have weakened immune systems. They are more susceptible to lower respiratory tract infections before surgery, while postoperatively they are at risk for ARDS. The other 50% of infants’ ARDS were cause for CPB. Children with cardiac disease may be particularly susceptible to deleterious CPB interactions induced by positive pressure ventilation. Surfactant dysfunction and inactivation are key contributors to the pathophysiology of ARDS by inducing areas of atelectasis and intrapulmonary shunting, which reduce lung volumes and compliance. Because failure to improve clinically over the first several days, particularly regarding oxygenation, predicts a complicated course and greater mortality risk, oxygenation improvement is particularly important for these infants [23].

The study population was strictly defined according to inclusion and exclusion criteria. Most infants with mild lung injury could be well managed with common treatment such as mechanical ventilation. As a clinical predictive tool, a PaO_2_/FiO_2_ threshold of 150 was used in this study. Surfactant would be given as rescue treatment once mechanical ventilation failed in improving the oxygenation. Oxygenation improvement was reported in previous study [13, 24, 25]. Unlike previous studies, a decrease in the duration of ventilation and total time of PICU could also be observed.

Preliminary exploratory analysis showed: infants younger than 3 months achieved faster recovery of pulmonary function. Age is a risk factor of lung damage during CPB [26]. Lung ischemia-reperfusion was more severe in the infants younger than 3 months, probably because of the combination of low antioxidant capacity and overproduction of reactive oxygen species. And the rescue surfactant treatment would help with the avoidance of further lung injury and the maintenance of intact alveolar barrier.

Surfactant treatment aims to instill a liquid-surfactant mixture directly into the lung airway tree. By a mathematical model of 3D lung structure [27], the surfactant liquid plug deposits a coating film on the airway wall and then splits unevenly at the bifurcation due to gravity. Calculation results show the neonatal lung is a well-mixed compartment, whereas the older is not. That may partly explain why infants younger than 3 months get more benefit of oxygenation than the older ones, and the spread efficiency and homogeneity of surfactant may be different in the two subgroups. Comparisons between infants over and under than 3 months may help to promote future clinical practice. Positive surfactant treatment may give young infants more benefit in earlier stage.

Single-centered data and institution-specific variables may influence the results. More data are necessary to better understand the risks and benefits of low-dose surfactant therapy in infants with moderate-to-severe ARDS after cardiac surgery, and a multi-center randomized controlled trial for this topic is also necessary.

## Conclusions

ARDS continues to be associated with prolonged mechanical ventilation in CHD infants undergoing cardiac surgery and CPB. There is an urgent need for therapies which could alter the outcome of the disease. This study has presented that low-dose surfactant therapy could be safely and effectively applied in the treatment of infants with ARDS, surfactant instillation has effects of improving oxygenation (OI, VI and PaO_2_/FiO_2_ ratio) and reducing the mechanical ventilation time and ICU time. There is justification to test the role of exogenous surfactant in cardiac surgical babies who develop ARDS in randomized controlled trials.

## Data Availability

The datasets used and analysed during the current study are available from the corresponding author on reasonable request.

## Abbreviations

ARDS: Acute respiratory distress syndrome
CHD: Congenital heart disease
CPB: Cardiopulmonary bypass
PICU: Pediatric cardiac surgical intensive care unit
PaO_2_/FiO_2_: Arterial oxygen concentration to the fraction of inspired oxygen
ECMO: Extracorporeal membrane oxygenation
RACHS-1: Risk adjustment congenital heart surgery-1
OI: Oxygenation index
VI: Ventilation index
CPAP: Continuous positive airway pressure
HFOV: High frequency oscillatory ventilation
CI: Confidence interval
